# Calcifediol: Mechanisms of Action

**DOI:** 10.3390/nu15204409

**Published:** 2023-10-17

**Authors:** Simone Donati, Gaia Palmini, Cinzia Aurilia, Irene Falsetti, Francesca Marini, Francesca Giusti, Teresa Iantomasi, Maria Luisa Brandi

**Affiliations:** 1 Department of Experimental and Clinical Biomedical Sciences, University of Florence, 50139 Florence, Italy; simone.donati@unifi.it (S.D.); gaia.palmini@unifi.it (G.P.); cinzia.aurilia@unifi.it (C.A.); irene.falsetti@unifi.it (I.F.); francesca.giusti@unifi.it (F.G.); teresa.iantomasi@unifi.it (T.I.); 2 Fondazione Italiana Ricerca Sulle Malattie dell’Osso (FIRMO Onlus), 50129 Florence, Italy; francesca.marini@unifi.it; 3 Donatello Bone Clinic, Villa Donatello Hospital, 50019 Sesto Fiorentino, Italy

**Keywords:** vitamin D, calcitriol, calcifediol, genomic actions, non-genomic actions, vitamin D receptor, membrane-associated rapid response to steroid, vitamin D deficiency

## Abstract

Due to its essential role in calcium and phosphate homeostasis, the secosteroid hormone calcitriol has received growing attention over the last few years. Calcitriol, like other steroid hormones, may function through both genomic and non-genomic mechanisms. In the traditional function, the interaction between the biologically active form of vitamin D and the vitamin D receptor (VDR) affects the transcription of thousands of genes by binding to repeated sequences present in their promoter region, named vitamin D-responsive elements (VDREs). Non-transcriptional effects, on the other hand, occur quickly and are unaffected by inhibitors of transcription and protein synthesis. Recently, calcifediol, the immediate precursor metabolite of calcitriol, has also been shown to bind to the VDR with weaker affinity than calcitriol, thus exerting gene-regulatory properties. Moreover, calcifediol may also trigger rapid non-genomic responses through its interaction with specific membrane vitamin D receptors. Membrane-associated VDR (mVDR) and protein disulfide isomerase family A member 3 (Pdia3) are the best-studied candidates for mediating these rapid responses to vitamin D metabolites. This paper provides an overview of the calcifediol-related mechanisms of action, which may help to better understand the vitamin D endocrine system and to identify new therapeutic targets that could be important for treating diseases closely associated with vitamin D deficiency.

## 1. Introduction

Vitamin D is found in virtually every form of life, from phytoplankton to humans, and is thought to be one of the oldest hormones on Earth [1]. Originally, vitamin D was defined as a vitamin because it could be obtained from food sources, mainly fatty fish, fish oils, dairy products, and some mushrooms.

Dietary vitamin D (vitamin D_2_ or D_3_) is normally absorbed by the small intestine along with other dietary fats [2]. The presence of fat in the intestinal lumen triggers the release of bile acids, which initiate emulsification and help to form lipid-containing micelles [3]. After ingestion, exogenous vitamin D is packaged into chylomicrons for transport to the liver. Part of the vitamin D contained in chylomicrons can be absorbed by adipose tissue and skeletal muscle [4]. Once the residual chylomicrons reach the liver, a vitamin D-binding protein (DBP) allows them to enter the liver cells, which then facilitates their transport to the various tissues that need them.

**Figure 1:**
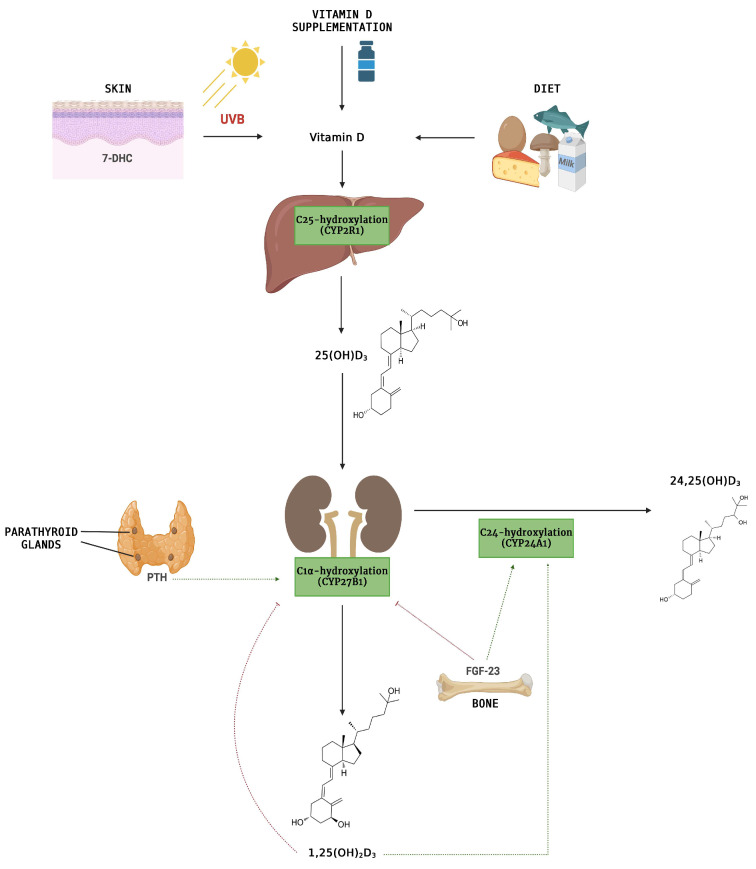
Schematic illustration of the vitamin D pathway: 25(OH)D_3_ metabolism. 7-DHC: 7-dehydrocholesterol; FGF-23: fibroblast growth factor 23; PTH: parathyroid hormone (PTH).

However, for humans, the photochemical production of 7-dehydrocholesterol (7-DHC) is the most important natural source of vitamin D that takes place in the basal layer of the epidermis in the skin [5]. In particular, the β-ring photodegradation of 7-DHC stimulated by solar ultraviolet type B (UVB) photon irradiation (around 280 to 320 nm) results in the production of previtamin D_3_ that undergoes a thermal isomerization to form vitamin D_3_ (or cholecalciferol) or alternatively photoconverted in two nonactive forms, such as tachysterol and lumisterol [6,7]. Following release from the cells, vitamin D_3_ enters circulation and is transported in the circulation bound to a vitamin D-binding protein (DBP) to storage tissues or the liver [8,9]. In the hepatocytes, vitamin D_3_ is rapidly hydroxylated at the C-25 position by 25-hydroxylase, a cytochrome P450 enzyme (mainly the CYP2R1), thus producing calcifediol or calcidiol (25(OH)D_3_). Once synthesized, DBP-bound 25(OH)D_3_ is secreted into the blood and transported to the kidney to obtain the biologically active form calcitriol (1α,25(OH)_2_D_3_) (Figure 1).

The average plasma half-life of 25(OH)D_3_ is approximately between 20 and 24 days, the highest compared to other vitamin D metabolites [10]. Therefore, the measurement of 25(OH)D_3_ is the gold standard method to determine the body’s vitamin D storage and status. To be exact, 25(OH)D_3_ is 1α-hydroxylated by another CYP450-dependent system activity (CYP27B1) in the mitochondria of the proximal convoluted tubule cells [11]. This reaction is closely regulated by blood phosphate and calcium levels through fibroblast growth factor 23 (FGF-23) and parathyroid hormone (PTH). Calcitriol is involved in the regulation of plasma concentrations of ionized calcium and phosphate by modulating their renal excretion, intestinal absorption, and calcium bone mobilization. If levels of 1α,25(OH)_2_D_3_ rise, calcitriol induces its degradation by stimulating the expression of 24-hydroxylase (CYP24A1), which is also responsible for the catabolism of 25(OH)D_3_ [8,10]. Generally, the 24-hydroxylation reaction is followed by several oxidation reactions and sometimes the conjugation with glucuronic acid to form many compounds that are excreted through the bile [8]. Under low serum calcium conditions, the parathyroid glands secrete PTH, which stimulates the expression of 1α-hydroxylase, leading to enhanced 1α,25(OH)_2_D_3_ activation [12]. PTH also inhibits 24-hydroxylase and induces the synthesis of FGF-23 by osteoclasts and osteocytes, which acts to reduce the expression of renal sodium phosphate transporter [13,14]. FGF-23 can also regulate vitamin D homeostasis by inhibiting renal expression of 1α-hydroxylase and inducing 24-hydroxylase, thereby reducing serum calcitriol levels, which in turn reduce serum calcium levels in hyperphosphatemic conditions [15].

The human population is currently experiencing a high prevalence of moderate to severe vitamin D deficiency, which has detrimental effects on musculoskeletal and extra-skeletal systems. There is a general but not unanimous consensus that 25(OH)D_3_ levels <20 ng/mL are inadequate for maintaining musculoskeletal health, leading to rickets in children, osteomalacia in adults, and secondary hyperparathyroidism, while no serum 25(OH)D_3_ concentration is recommended for extra-skeletal health outcomes [16].

Based on more than 500 studies worldwide, 88% of the world’s population have serum 25(OH)D_3_ levels <30 ng/mL, approximately 37% have values below 20 ng/mL, and about 7% have levels below 12 ng/mL [17]. Moreover, there are some groups or regions whose prevalence of vitamin D deficiency is higher.

The causes of vitamin D deficiency are varied and include limited sunlight exposure, sunscreen use, inadequate intake of foods containing vitamin D, dark skin pigmentation, patients with intestinal malabsorption syndromes, genetic diseases of vitamin D metabolizing enzymes or the vitamin D receptor (VDR), drugs that can interfere with the vitamin D absorption or metabolism, hepatic disease, obesity, aging, and renal disease [18,19]. On this latter aspect, approximately 80% of chronic kidney disease (CKD) patients have a high prevalence of vitamin D deficiency/insufficiency. Considering the extra-renal expression of 1α-hydroxylase, increasing interest was placed on vitamin D supplementation, including vitamin D_2_, vitamin D_3_, and 25(OH)D_3_, highlighting the importance of peripheral production of calcitriol. Despite the Italian Society of Nephrology (SIN) having already published a series of guidelines about vitamin D in CKD patients in 2016, indicating to supplement patients with CKD stages 3–5 and serum 25(OH)D_3_ concentration <30 ng/mL, the risk of toxicity should always be monitored, including adverse health effects associated with hypervitaminosis D, such as hyperphosphatasemia and hypercalcemia [20].

There are only a limited number of strategies to prevent or correct hypovitaminosis D. Increasing sun time exposure could, on the one hand, increase the skin synthesis of vitamin D, but on the other hand, considering that UVB is also photocarcinogen, it is not advisable to recommend a greater time exposure to sun, especially for those people with higher sensitivity to DNA [21]. In this light, it is well established that most dermatologists recommend avoiding long-term exposure to UVB light to prevent the development of long-term consequences, such as skin cancer. Furthermore, there are other limitations to implementing this strategy in real life, such as climatic circumstances, skin color, lifestyle, and cultural and religious habits [22]. Another option could be to increase the consumption of vitamin D-containing foods, but this is not feasible because there are no sufficient oils from fish in the ocean, one of the major dietary sources of vitamin D, to correct vitamin D deficiency status around the world. A good strategy is the intake of vitamin D- or calcifediol-fortified foods, and it has been successfully implemented in many northern European countries such as Finland [23]. However, this option becomes less effective in populations with a great variety of food preferences. Since the discovery of vitamin D, vitamin D supplementation has been a widely used valid strategy to prevent or correct such deficiency. The preferred compound globally used is vitamin D_3_, even though vitamin D_2_ is the vitamin D supplement of choice in the USA for the Steenbock patent and India because cholecalciferol is from animal origin [24,25]. Apart from these metabolites, even calcifediol has been considered as an oral vitamin D supplement to help people who are more likely to acquire hypovitaminosis D [26]. Based on Italian guidelines (https://www.aifa.gov.it/en/nota-96 (accessed on 29 September 2023)), the recommended dosage regimens for calcifediol and vitamin D_3_ depend on the individual’s vitamin D status. In particular, an intake of 266 μg of calcifediol twice per month is recommended when serum 25(OH)D_3_ concentration is lower than 30 nmol/L. In a range of serum 25(OH)D_3_ concentration between 30 and 50 nmol/L, the recommended dosage for calcifediol is 266 μg once a month, while no vitamin D supplementation is generally not needed for those people with vitamin D blood levels above 50 nmol/L.

The main function of vitamin D is to regulate calcium–phosphate homeostasis, thereby contributing to maintaining proper bone health. The calciotropic hormone calcitriol functions as a steroid by binding to the intracellular VDR [27], a member of the steroid nuclear receptor superfamily, which includes retinoic acid, thyroid hormone, adrenal steroids, and sex hormones. This binding results in the modulation (suppression or activation) of gene expression [10,28,29,30,31].

Given that the presence of VDR is well documented in the cells of various organs, it has been established that the proper functioning of musculoskeletal, nervous, cardiovascular, and immune systems is strongly dependent on vitamin D [6,28]. Therefore, vitamin D deficiency status was not only described as a risk factor associated with the occurrence of rickets or osteoporosis [32] but also with the altered function of the immune system and the ability to mobilize a response to invading pathogens, including influenza and SARS-CoV-2 [33]. Over the last decade, it has been reported that vitamin D has a role in brain development and function [34]. Furthermore, it was suggested that levels of serum 25(OH)D_3_ in the range of 40 to 60 ng/mL are beneficial, reducing the occurrence and aggressiveness of several types of cancer [35] and showing cardioprotective [36] and neuroprotective [37] properties.

The biological effects of 1α,25(OH)_2_D_3_, like other steroid hormones, are mediated via both genomic and non-genomic mechanisms. Recently, 25(OH)D_3_ has been demonstrated to affect the expression of genes as well as to trigger non-genomic responses through its interaction with distinct membrane-associated, rapid-response steroid-binding receptors (MAARS) [22,38,39,40]. In this narrative review, we will provide an overview of the calcifediol-related mechanisms of action, which may help to better understand the vitamin D endocrine system and to identify new therapeutic targets that could be important for treating diseases closely associated with vitamin D deficiency.

## 2. Vitamin D Classical Actions: Regulation of Calcium and Phosphate Homeostasis

Calcitriol serves as one of the primary regulators of serum calcium levels within the optimal range by directly acting on three target tissues. Moreover, 1α,25(OH)_2_D_3_ suppresses parathyroid gene expression and parathyroid cell proliferation via the VDR, emphasizing its direct action on enhancing serum calcium levels [41].

One of the target organs is the intestine, where 1α,25(OH)_2_D_3_ stimulates intestinal calcium absorption [2]. This effect depends on the presence of dietary calcium, its intestinal solubility, and intestinal absorptive capacity as a result of the balance between transcellular and paracellular intestinal absorption. Transcellular transport involves three parts: calcium entry through specific calcium channels in the brush border membrane, intracellular transport through calbindin, and active calcium transport to the bloodstream on the basolateral surface, mainly via specific carriers.

The second organ is the kidney, in which both 1α,25(OH)_2_D_3_ and PTH promotes calcium reabsorption in the distal renal tubules [15]. Calcitriol affects (I) calcium entry through the apical membrane, (II) calcium diffusion mediated by calbamicin, and (III) active transport across the basolateral membrane. In addition, vitamin D inhibits phosphate reabsorption directly by inducing α-klotho and indirectly by enhancing FGF-23 osteocyte expression.

In the bones, 1α,25(OH)_2_D_3_ promotes the release of calcium from bone in a process requiring PTH [42]. In particular, the PTH-dependent calcitriol activation, as a consequence of the decreased serum calcium levels, stimulates the VDR-mediated formation and differentiation of osteoclasts. This activation results in the increase of skeletal calcium mobilization by stimulating the receptor activator for nuclear factor kappa-B ligand (RANKL) secretion, which, in turn, is a potent stimulator of osteoclastogenesis and bone resorption [43]. Moreover, vitamin D suppresses mineralization by enhancing pyrophosphate levels and osteopontin [44]. Therefore, vitamin D deficiency can lead to inadequate mineralization of the skeleton, thereby contributing to osteoporosis and fractures [45,46].

## 3. Vitamin D Molecular Actions

### 3.1. Genomic Response of Calcitriol

The secosteroid 1α,25(OH)_2_D_3_ is a calciotropic hormone that functions as a steroid molecule by its interaction with the intracellular VDR [27]. The VDR gene, which consists of eight coding exons, is found in fish, birds, and mammals and encodes a protein of 427 amino acid residues [47,48]. The two functional domains of the VDR are the conserved NH_2_-terminal DNA-binding domain named DBD and a highly variable COOH-terminal ligand-binding domain named LBD [22]. Calcitriol binding triggers conformational change, resulting in the dissociation of the repressor protein, thus promoting the dimerization of VDR to form either homodimers or heterodimers with retinoid X receptor (RXR) [10]. Once dimerized, both complexes (homo- and heterodimeric) bind to repeated sequences generally positioned in the physical proximity of transcription start site specifical of vitamin D-target genes named vitamin-D-responsive elements (VDREs) to regulate negatively or positively their expression [49]. Association of 1α,25(OH)_2_D_3_:VDR:RXR with the VDREs leads to the recruitment of coactivators that have histone acetylase activity, affecting the binding affinity of hystone proteins and DNA [50]. Furthermore, this large complex functions by recruiting RNA polymerase II enzyme to the transcription start site, thereby regulating the transcription of different genes involved in the control of calcium homeostasis (i.e., alkaline phosphatase (ALP), cytochrome P450 family (CYP450) 24 (CYP24), type I collagen (COL1A1), PTH, osteopontin, osteocalcin (bone gamma-carboxyglutamate protein (BGLAP), and transient receptor potential vanilloid type family member 6 (TRPV6)) [10,28,29,30,31,50]. The increase or decrease in protein expression levels caused by gene transcription regulation from steroid hormones is the result of their genomic actions. The timing of these actions is not immediate but rather delayed, as they take time for newly synthesized proteins and processing.

The totality of the above-mentioned transcriptional complexes defines the sensitivity and specificity of the various steroid hormones, including vitamin D [50]. In particular, the physiological response specificity is determined by the 1α,25(OH)_2_D_3_. The genetic specificity is guided by the VDRE. The cell or tissue specificity of the response depends on the different proteins recruited following the binding of 1α,25(OH)_2_D_3_-VDR-RXR to the VDRE. Finally, the biological response is caused by the activity of the vitamin D-responsive gene product.

#### 25(OH)D_3_-Related Genomic Responses

Calcitriol is the only high-affinity (KD = 0.1 nM) ligand of the VDR [51], whereas the receptor affinity for 25(OH)D_3_ is 100- to 1000 times lower [52]. This discrepancy could be explained by the fact that 25(OH)D_3_ is missing a 1-OH-group, while generally the specific binding within the VDR’s LBD pocket is achieved through interactions between a pair of polar amino acids and the three hydroxyl groups of 1,25(OH)_2_D_3_ (i.e., R274 and S237 bind the 1-OH-group, Y143 and S278 the 3-OH-group, and H305 and H397 the 25-OH-group) [53,54]. Nevertheless, the serum levels of 25(OH)D_3_ (50–250 nM) are 1000-fold higher compared to those of 1,25(OH)_2_D_3_ (0.05–0.15 nM), and therefore its levels should be enough for effective binding and to act as an agonistic VDR ligand [55,56].

As high levels of 25(OH)D_3_ are found in serum, blood cells are one the best experimental systems for investigating a possible gene regulatory function of 25(OH)D_3_. A study carried out by Hanel et al. [40] demonstrated that higher concentrations of 25(OH)D_3_, such as 1 μM and 10 μM nM, affect gene expression in peripheral blood mononuclear cells (PBMCs) isolated from five healthy individuals in a comparable way observed with 10 nM 1,25(OH)_2_D_3_. Among the 398 targets of 1 μM 25(OH)D_3_, approximately 86% also responded to 1,25(OH)_2_D_3_ in PBMCs, while the rate for the 477 target genes of 10 μM 25(OH)D_3_ was 78.0%. However, only two genes, MYLIP and ABCG1, both involved in cholesterol transport, were specifically regulated by 10 μM 25(OH)D_3_.

In a subsequent study from the same research group [39], the authors found that the vitamin D metabolites 25(OH)D_3_ and 25(OH)D_2_ are equally able to directly modulate vitamin D-target gene expression, as well as 1,25(OH)_2_D_3_, the most potent VDR ligand, even though higher concentrations of 300 nM are required to observe these 25(OH)D-associated genomic responses.

Overall, these findings suggest that 25(OH)D_3_ within the physiological range (100–250 nM) does not affect gene expression, even though higher concentrations trigger such responses, although it is not to be excluded that the enzymatic formation of 1,25(OH)_2_D_3_ could partially contribute to modulate the transcriptome of PBMCs. This option cannot be excluded because these cells show reduced but significant expression levels of the CYP27B1 gene.

A similar expression pattern was also found in the prostate cancer cell line LNCaP [57].

The intriguing finding is that the metallothionein 2A gene was found to be a distinct target for 1,25(OH)_2_D_3_ but not for 25(OH)D_3_ [58]. Moreover, it is conceivable that the transcription factors encoded by genes activated by 1,25(OH)_2_D_3_ could, in turn, modulate the expression of additional gene sets [59], which could be defined as a secondary genomic response, even though this mechanism must still be defined even for 25(OH)D_3_.

### 3.2. Rapid Non-Genomic Actions

In 1942, Hans Selye described that progesterone showed an anesthetic effect immediately after peritoneum injection in rodents, which was different from what was observed with regard to its primary function, which occurred only within hours after its administration [60]. This is the first report of non-genomic actions mediated by steroid molecules. Then, Spach and Streeten showed that Na^+^ ions changed within a few minutes after the administration of aldosterone in dog erythrocytes, providing novel compelling evidence for the aldosterone-related non-genomic effects precisely for the absence of nuclei in these cells [61]. However, these non-transcriptional effects were not known until recently, when different rapid responses were recognized for various steroid hormones, including 1α,25(OH)_2_D_3_ [62].

In spite of the genomic counterpart, non-genomic rapid effects can be observed within seconds or minutes after stimulation, without the need for activation of gene expression and de novo subsequent protein synthesis. In this regard, these rapid mechanisms are not susceptible to molecules that inhibit the genomic effects, such as cycloheximide or actinomycin D, and also occur in response to steroids coupled to large proteins that do not allow their entry into the cells [29].

#### 3.2.1. Membrane-Associated Proteins and Targets for Vitamin D-Mediated Non-Genomic Responses

The idea of the existence of alternative non-genomic pathways activated by vitamin D was conceived by the pioneering studies of Nemere and colleagues in 1984 [63], who observed that 1α,25(OH)_2_D_3_ induced a rapid influx of intracellular calcium either by promoting its release from intracellular compartments or by improving the intestinal absorption in vascularly perfused duodenum from normal, vitamin D-replete chicks. This rapid influx of calcium within 14 min in response to 1α,25(OH)_2_D_3_ in a mechanism of genome activation- and protein synthesis-independent was named transcaltachia.

According to the time frame and the insensitivity of non-genomic responses to transcription and translation inhibitors, it was supposed that 1α,25(OH)_2_D_3_ triggers non-transcriptional effects by interacting with intracellular and membrane-associated macromolecules. This idea was corroborated by the observation that several secondary messengers were produced in response to 1α,25(OH)_2_D_3_, such as phosphatidylinositol (3,4,5)-trisphosphate (PIP3), calcium, and cyclic AMP (cAMP), culminating in a successful downstream activation of different protein kinases (PKs) (PKC, mitogen-activated protein (MAP) kinases, calcium/calmodulin-dependent protein kinase II gamma (CaMKIIG), and Src) [64,65,66,67,68]. Moreover, this secosteroid was also described to mediate the opening of calcium, chloride, and phosphate ion channels.

Several studies demonstrated that 1α,25(OH)_2_D_3_ interacts with the membrane isoform VDR (mVDR) associated with caveolin-1 (CAV1) and non-classical MAARS, resulting in fast non-genomic responses to vitamin D [69,70,71].

The existence of a distinct mVDR serving as the mediator of vitamin D-associated non-genomic signaling was described for the first time by the early studies of Norman et al. [69,70]. In this study, the authors observed that 1α,25(OH)_2_D_3_ in its 6-s-cis configuration was able to trigger rapid non-transcriptional responses, while the 6-s-trans configuration mediated the genomic responses.

Interestingly, the activation of at least some 1α,25(OH)_2_D_3_-induced non-genomic effects was displayed to be mVDR-dependent, such as the activation of SRC proto-oncogene non-receptor tyrosine kinase, and the regulation of several signaling pathways, such as Notch [72,73,74], sonic hedgehog (Shh) [75,76,77,78,79,80], and Wnt [81,82,83,84].

Another remarkable non-genomic activity of 1α,25(OH)_2_D_3_ was observed on models of adenosine diphosphate (ADP)- or collagen-induced platelet aggregation. Here, the authors [85] found that 1α,25(OH)_2_D_3_ had an inhibitory effect on platelet aggregation, which varied according to the state of diabetes. In particular, glycemic control was inversely and significantly associated with high platelet aggregation and reduced 25(OH)D_3_ levels. The absence of a nucleus and the presence of VDR in platelets indicated that this mechanism should be the outcome of the activation of non-genomic pathways.

Baran and colleagues [86] showed that 1α,25(OH)_2_D_3_ not only induced a rapid opening of calcium channels but also promoted a rapid activation of phospholipase C (PLC) in ROS 24/1 cells lacking VDR, indicating that these effects do not require the VDR involvement. The same finding was also validated in cultured costochondral chondrocytes derived from VDR^−/−^ mice, where Boyan et al. [87] found that 1α,25(OH)_2_D_3_ was involved in the regulation of PKC activity.

This suggests that in addition to the presence of mVDR, the existence of other membrane receptors together with vitamin D could be essential for rapid non-genomic in response to 1α,25(OH)_2_D_3_.

One of the best characterized membrane-associated proteins was described in the above-mentioned study of Nemere et al. [63], who originally reported not only the ability of 1α,25(OH)_2_D_3_ to induce transcaltachia but also the purification of a plasmalemma receptor able to bind to the radiolabeled 1α,25(OH)_2_D_3_, with a KD-value of 0.72 nM. Later, this protein was termed protein disulfide isomerase family A member 3 (Pdia3) [88,89]. This protein interacts with two known molecular chaperones, calreticulin (CALR) and calnexin (CANX), playing a crucial role in the correct folding and export of newly synthesized glycoproteins [90]. Aside from cell membrane localization, Pdia 3 was also identified in mitochondria, cytosol, and nucleus, suggesting its involvement in different biological functions, such as cell protection from the ROS-induced damaging effects and the prevention of disorders associated with the accumulation of misfolded proteins [91]. The interaction between Pdia3 and 1α,25(OH)_2_D_3_ could also be an important protective mechanism against UV-driven DNA damage [92]. This response was associated with the activation of VDR-independent of the PKC signaling transduction pathway [93] and a rapid rise in intracellular calcium concentrations [94]. Further investigations have demonstrated that Pdia3-1α,25(OH)_2_D_3_ binding could be involved in the activation of PLA2 via PLA2-activating protein (PLAA) [71], MAPK1, and MAPK2 via the regulation of calcium/calmodulin-dependent protein kinase II gamma (CaMKIIG), PLA2, PLC, and PKC [65,95], and Wnt family member 5A (Wnt5A) [96].

Collectively, these findings pointed out that Pdia3 is essential for mediating the rapid non-genomics in response to vitamin D. Furthermore, some groups also suggested the nuclear localization of Pdia3, even though there is an ongoing debate about whether the secosteroid 1α,25(OH)_2_D_3_ could influence Pdia3 translocation into the nucleus and if the latter could act as a transcription factor or could promote the recruiting of other transcription factors [97].

#### 3.2.2. 25(OH)D_3_-Related Non-Genomic Responses

Calcifediol was, for a long time, recognized to be only a prohormone, a precursor of the biologically active form of vitamin D activated by the 1α-hydroxylation that takes place in proximal convoluted tubules of the kidney. As previously discussed in this review, recent evidence demonstrated that 25(OH)D_3_ interacts with VDR with a lower affinity (about 50 times) respect than 1α,25(OH)_2_D_3_.

Given the ability of 25(OH)D_3_ to join with the VDR, we [38] supposed that 25(OH)D_3_ could exert a fast non-genomic response, such as a sustained increase of intracellular calcium concentrations, in mesenchymal stem cells derived from human adipose tissue (hADMSCs), earlier depicted as an excellent cell model system for investigating the secosteroid hormone 1α,25(OH)_2_D_3_-related effects. We found out for the first time that 25(OH)D_3_ increases intracellular calcium levels in hADMSCs at much higher concentrations generally present in the human body (nanomolar range). As previously reported for the 25(OH)D_3_-related genomic effects, this could be the result of the reduced binding affinity of this vitamin D metabolite for VDR.

This finding was consistent with the non-genomic rapid effects of 25(OH)D_3_ in human spermatozoa, where Blomberg Jensen, M. et al. [98,99] showed that the immediate precursor of 1α,25(OH)_2_D_3_ was incapable of affecting calcium levels within the subnanomolar range, while a sustained but delayed intracellular calcium concentration rise was observed in human spermatozoa in response to of 25(OH)D_3_ at higher concentrations.

Interestingly, 25(OH)D_3_ was also found to regulate lipogenesis via a VDR-independent non-genomic effect that involved the processing and degradation of sterol regulatory element-binding protein (SREBP) cleavage-activating protein (SCAP) in the endoplasmic reticulum [100]. This intriguing observation could have a physiological significance in reducing the risk of metabolic syndrome-associated complications, in which an inverse correlation between serum 25(OH)D_3_ levels and severity was observed.

The disruption of endothelial integrity and the intensification of vascular ruptures have been shown to be implicated in pathogenic conditions, which can be prevented by adequate intake of vitamin D and its derivates. In addition to the canonical transcription-mediated vitamin D pathway, data showed that vitamin D_3_-related non-genomic responses could play an essential in maintaining both epithelial and endothelial cell stability [101]. Thus, vitamin D deficiency could impair the body’s protective systems, resulting in the leakage of vascular fluid and worsening infections, ultimately leading to septicaemia [101]. Hence, the fast, non-transcriptional functions mediated by vitamin D might aid in the resolution of inflammation and infections and keep the endothelial junction integrity. As a result, deficiency in vitamin D could increase the vulnerability and the severity of infections and chronic diseases, which can lead to increased complications incidence rate and early death [102].

## 4. Discussion

The vitamin D endocrine system has been recognized as an essential part of the control of body calcium and phosphate levels, which facilitate adequate muscle performance, bone growth, and mineralization. These activities result from the activation of the VDR in intestinal, bone, and renal tissues by 1α,25(OH)_2_D_3_, primarily through direct interactions between 1α,25(OH)_2_D_3_-activated VDR and chromatin. Furthermore, it is now well-known that 1α,25(OH)_2_D_3_ has biological effects that do not involve gene transcription, which include the regulation of intracellular calcium as well as the activation of several signaling pathways via PKs and phosphatases [50].

More recently, studies showed that 25(OH)D_3_, the direct precursor of 1α,25(OH)_2_D_3_, can not only able to activate genomic responses but also modulate rapid non-genomic actions.

As described in this review, although the affinity of VDR for 25(OH)D_3_ is 100- to 1000-times lower compared to 1α,25(OH)_2_D_3_ [52], due both to a larger ligand-binding pocket and the absence of a 1-OH-group in the major circulating form of vitamin D, 25(OH)D_3_ is able to affect gene expression at concentrations higher than the physiological range. Moreover, a similar expression gene pattern was found between 25(OH)D_3_ and 1α,25(OH)_2_D_3_ either in the transcriptome of PBMCs and LNCaP [39,40,57]. Recently, it has been demonstrated that 1,25(OH)_2_D_3_ exerts regulatory effects on microRNA expression and long non-coding RNAs, potentially serving as a potential anticancer mechanism [103,104]. In this regard, no evidence has been established for the 25(OH)D_3_-induced regulation of these classes of non-coding RNA so far.

Furthermore, there is increasing evidence that 25(OH)D_3_ can also induce rapid, non-genomic activities, although their impact on physiological processes has not been clarified yet. In this regard, one of the possible physiological implications of the 25(OH)D_3_-mediated rapid non-genomic effect is the direct control of lipogenesis via a VDR-independent mechanism that involves the degradation of SCAP in the endoplasmic reticulum [100]. These findings could provide important clinical implications for alternative pathways activated by 25(OH)D_3_ to reduce the risk of complications associated with metabolic syndromes. Another clinical implication of 25(OH)D_3_-induced signaling pathways is the maintenance of epithelial and endothelial cell stability, which could be relevant for reducing the susceptibility and severity of chronic diseases associated with reduced levels of vitamin D. As reported in our previous study, 25(OH)D_3_ is able to increase intracellular calcium levels in hADMSCs here, similarly to what observed for the biologically active form of vitamin D. The rapid 1α,25(OH)_2_D_3_-induced stimulation of intestinal calcium is one of the most noticeable non-genomic effects of vitamin D, which serves to regulate the calcium level in the body [6,22]. However, additional studies are needed to provide conclusive evidence of the implications of such rapid action on meal feeding and calcium absorption physiology. Moreover, there is a growing body of evidence indicating that the rapid and non-genomic responses have the potential to both positively and negatively impact genomic functions [22]. This finding was demonstrated for 1α,25(OH)_2_D_3_, revealing that this secosteroid hormone can activate different signaling molecules involved in several signaling pathways, such as PI3K, PLC, and PLA2, thus affecting gene expression via primary regulatory elements of gene expression or using activated as a substrate. Furthermore, this interplay could influence both the effectiveness and the strength of gene regulation.

**Figure 2:**
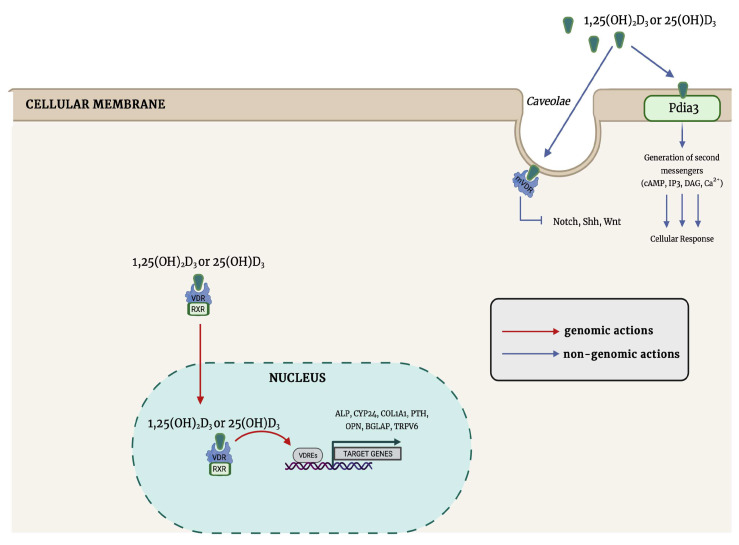
The proposed vitamin D-related genomic and non-genomic signaling pathways. Abbreviations: VDR: vitamin D receptor; RXR: retinoid X receptor; VDRE: vitamin D_3_ response elements; Pdia3: protein disulfide isomerase family A member 3; mVDR: membrane-bound VDR; Shh: Sonic hedgehog; WNT: Wingless/Integrated; DAG: diacylglycerol; IP3: inositol trisphosphate; cAMP: cyclic AMP; ALP: alkaline phosphatase; CYP24: cytochrome P450 family; COL1A1: type I collagen; PTH: parathyroid hormone; OPN: osteopontin; BGLAP: bone gamma-carboxyglutamate protein; TRPV6: transient receptor potential vanilloid type family member 6.

The field of vitamin D has made considerable advancements over the last years, expanding its understanding beyond the previously reported role in calcium regulation and rickets and osteomalacia prevention, respectively, in children and adults. Unfortunately, vitamin D-mediated non-genomic mechanisms are still not fully understood, and further studies are needed to reveal their functions in physiology, pathology, and clinical application potential. Although few studies are available in the literature on this field, a growing body of evidence has shown that 25(OH)D_3_ is an agonistic VDR ligand providing elucidation both for the direct gene regulatory properties and membrane non-genomic responses that have been defined for this vitamin D metabolite (Figure 2). Therefore, studies aiming to improve our understanding of the vitamin D endocrine system could be crucial to developing approaches for the prevention and treatment of chronic pathologies linked with suboptimal vitamin D status.

## Data Availability

Not applicable.
